# *Asteraceae* Seeds as Alternative Ingredients in a Fibre-Rich Diet: Protein Quality and Metabolic Effects in Rats

**DOI:** 10.3390/molecules28073275

**Published:** 2023-04-06

**Authors:** Jarosław Koza, Adam Jurgoński

**Affiliations:** 1 Department of Gastroenterology and Nutrition Disorders, Faculty of Health Sciences, Collegium Medicum in Bydgoszcz, Nicolaus Copernicus University in Toruń, 87-100 Toruń, Poland; 2 Department of Biological Function of Food, Institute of Animal Reproduction and Food Research, Polish Academy of Sciences, 10-748 Olsztyn, Poland

**Keywords:** digestibility, fibre, herbs, lipid metabolism, milk thistle, nitrogen balance, pot marigold, protein, seeds

## Abstract

We verified whether milk thistle seeds and pot marigold seeds provided valuable components for a fibre-rich diet and how their addition affected body composition, nitrogen balance and lipid metabolism in rats. Growing rats were fed a control diet (5% fibre) or three fibre-rich diets (24% fibre), which contained cellulose as the sole source of fibre (24%; positive control), milk thistle seeds (32%) or pot marigold seeds (39%). All diets were balanced in macronutrients, including total protein content (9%), which was half of the amount recommended for rats to maximise protein absorption and utilisation, and the ratio of plant protein to animal protein (approx. 1:1). After 4 weeks, dietary pot marigold seeds reduced body weight gain, which translated into lower gains of body fat and lean mass in rats (all at *p* ≤ 0.05). Protein digestibility differed among individual fibre-rich diets (*p* ≤ 0.05), with the lowest result having been recorded for dietary pot marigold seeds (73%), followed by dietary milk thistle seeds (78%), and the highest result having been recorded for dietary soybean protein isolate (control protein source, 89%). Nitrogen retention was higher with dietary soybean protein isolate (53%) and dietary milk thistle seeds (47%) than with dietary pot marigold seeds (38%) (*p* ≤ 0.05). In the caecal digesta, the concentrations of the major short-chain fatty acids were almost or >2-fold higher after dietary milk thistle seeds and pot marigold seeds than after the positive control diet (all at *p* ≤ 0.05). Dietary pot marigold seeds enlarged the liver and increased the plasma activities of liver enzymes but reduced hepatic lipid contents (all at *p* ≤ 0.05). Certain *Asteraceae* seeds provide components of varied nutritional quality, with milk thistle seeds being a relatively good source of protein and both types of seeds being a source of fermentable fibre. Pot marigold seeds have potential anti-obesogenic effects, but with the risk of damaging internal organs.

## 1. Introduction

Many medicinal plants grown on herbal plantations represent the family *Asteraceae*, including milk thistle (*Silybum marianum* L.) and pot marigold (*Calendula officinalis* L.). Milk thistle is currently one of the most popular herb species due to its seeds, which contain approximately 4% of a characteristic isomeric mixture of flavonolignans collectively referred to as silymarin [1,2,3]. Despite low bioavailability, flavonolignans extracted from milk thistle seeds (MTSs) are well-recognised hepatoprotective compounds, and they are sold for this reason as dietary supplements. In addition, many other bioactivities, such as antioxidant or anti-inflammatory activity, are also ascribed to MTSs [4,5]. However, MTSs are rich in nutrients, including protein, fat and fibre (approx. 20%, 20% and 42%, respectively), indicating that they may also be considered for nutritional purposes [6,7,8]. Leucine, valine and lysine are the most abundant essential amino acids found in MTSs [7,8], which are also rich in unsaturated fatty acids, especially linoleic and oleic acids, and other lipid-soluble compounds, such as phytosterols and *α*-tocopherol [9,10,11].

Another potential source of nutrients and bioactive compounds may be the seeds of pot marigold (*Calendula officinalis* L.), naturally occurring in the form of achenes. However, the chemical composition of pot marigold seeds (PMSs) has not been fully recognised thus far, whereas the vegetative parts of this plant, especially the flowers, are the most valuable herbal and cosmetic raw material of pot marigold [12]. Pot marigold extracts from flowers and leaves have broad beneficial effects, of which anti-inflammatory and wound-healing properties are the most documented [13,14]. In contrast to milk thistle, in pot marigold, there is not one specific group of compounds responsible for its beneficial effects, because it contains many groups, including triterpenes, flavonoids, carotenoids, sesquiterpenes and arabinogalactan polysaccharides [12,14]. Moreover, the flowers of pot marigold are added to salads or used as a saffron substitute or food colouring [14], and pot marigold flower extract encapsulated in microparticles has also recently been under consideration as a food additive [15]. PMSs contain up to 20% fat that is rich in calendic acid [16,17], which represents conjugated isomers of *α*-linolenic acid with many potential bioactivities [18].

There is a need to identify new, alternative sources of nutrients, including high-quality protein of plant origin and other components that are usually deficient in the diet, such as fibres or polyunsaturated fatty acids. The need is especially important in the contexts of malnutrition, which is present in some developing countries, and highly processed food, which is co-responsible for the worldwide pandemic of obesity and diet-related diseases [19,20]. Certain herb seeds of the family *Asteraceae* additionally rich in bioactive compounds are candidates that may diversify and improve the daily diet. However, nutritional research on the use of *Asteraceae* seeds as alternative sources of nutrients and other dietary components is lacking. Therefore, the aim of the present study was to verify whether MTSs and PMSs provide quality dietary components and how their regular consumption affects the body. An important part of the present study was to determine the protein value of these seeds by comparing it to a well-known plant counterpart, namely, soybean protein. For these purposes, a fibre-rich (FR) diet containing MTSs or PMSs was designed to determine their effects on body composition, nitrogen excretion pattern, internal organ function (distal intestine, liver and kidneys) and lipid metabolism in experimental rats. All diets were balanced in terms of macronutrients, including total protein content (9%), which was half of the amount recommended for rats to maximise protein absorption and utilisation, and the ratio of plant protein to animal protein (approx. 1:1). We hypothesised that including the selected herb seeds of the family *Asteraceae* in an FR diet could change the nutritional value of the diet and provide additional health benefits depending on the type of seeds and their chemical composition.

## 2. Results

### 2.1. Dietary Intake, Body Weight and Body Composition of Rats

**Figure 1:**
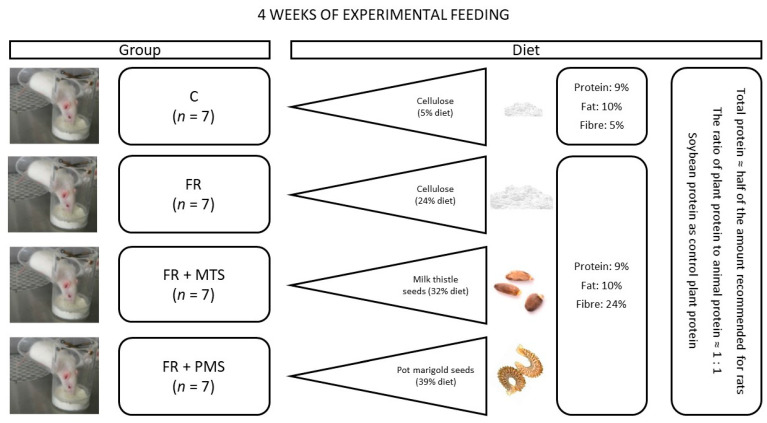
Scheme of experimental design. C, control group fed a diet containing soy protein isolate; FR, positive control group fed a fibre-rich diet containing soy protein isolate; FR + MTS, experimental group fed an FR diet containing milk thistle seeds; FR + PMS, experimental group fed an FR diet containing pot marigold seeds.

After 4 weeks of experimental feeding, the rats in the dietary FR groups (positive control) had lower final body weight and body weight gain than the rats in the control (C) group. Both the FR and C groups were fed diets containing comparable amounts of protein (9%) and fat (10%), but the diets considerably differed in the amount of fibre (24% vs. 5%, respectively). Details of the experimental design are shown in Figure 1 (please also see Section 4, Materials and Methods for more details). The FR group had dietary intake comparable to that of the FR + MTS and FR + PMS groups (Table 1), in which the FR diet fed to the FR group was modified by including MTSs and PMSs, respectively. However, dietary intake was lower in the FR + PMS group than in the FR + MTS group, which indicates that the diet fed to the FR + MTS group was less palatable. Compared with the FR group, the final body weight and body weight gain were lower in the FR + PMS group, which also translated into lower gains of body fat mass, lean mass and free fluids in this group. However, the initial and final percentages of body fat mass, lean mass and free fluids did not differ between the C and FR groups or among the FR groups.

### 2.2. Nitrogen Balance, Protein Digestibility and Nitrogen Retention in Rats

After a 5-day nitrogen balance period, the total nitrogen intake did not differ between the C and FR groups or among the individual FR groups (Table 2), all of which had comparable protein ratio (plant/animal ≈ 1) and protein content (9%) in the diet. The faecal and urine nitrogen contents were higher in the FR group than in the C group. In the FR + MTS and FR + PMS groups, which contained milk thistle seed protein and pot marigold seed protein in the diet, respectively, instead of soy protein, used as a plant protein source in the C and FR groups, the faecal nitrogen content was approximately twice as high as that in the FR group. The faecal nitrogen content calculated as the percentage of nitrogen intake was also approximately two times higher in the FR + MTS and FR + PMS groups than in the FR group. In addition, the faecal nitrogen content calculated as the percentage of nitrogen intake was higher in the FR + PMS group than in the FR + MTS group.

The results of the nitrogen balance were translated into differences in apparent protein digestibility and apparent nitrogen retention expressed in percentages. The apparent protein digestibility and nitrogen retention were lower in the FR group (89% and 53%, respectively) than in the C group (91% and 62%, respectively). The total amount of protein digested during the 5-day balance period was lower in the FR + PMS group than in the FR group. In addition, the apparent protein digestibility differed among the individual FR groups, with the highest result having been recorded in the FR group (89%) followed by the FR + MTS group (78%) and the FR + PMS group (73%). The total amount of nitrogen retained during the 5-day balance period and the apparent nitrogen retention were lower in the FR + PMS group than in the other FR groups.

### 2.3. Caecal Metabolism in Rats after 4 Weeks of Feeding

A fibre-rich diet fed to the FR group and the inclusion of MTSs or PMSs, which became the main source of dietary fibre in the FR + MTS or FR + PMS group, respectively, significantly affected the caecal metabolism in rats (Table 3). Importantly, all FR diets were matched in their fibre content (24%). Both the empty caecal mass and caecal digesta mass were higher in the FR group than in the C group. In addition, the caecal digesta mass was higher in the FR + PMS group than in the FR and FR + MTS groups. In the caecal digesta, all individual short-chain fatty acid (SCFA) concentrations and the total SCFA concentration, as the main fermentative metabolites of dietary fibre, were almost or more than two times lower in the FR group than in the C group. This considerable difference was due to the reduced dietary level of corn starch, including resistant starch, at the expense of which cellulose was included to create the FR diet.

In contrast, the total and major individual SCFA concentrations (acetate, propionate and butyrate) in the FR + MTS and FR + PMS groups were twice as high as or higher than those in the FR group. Moreover, the propionate concentration was higher in the FR + MTS group than in the FR + PMS group. The percentage of major SCFAs, including acetate, propionate and butyrate, also differed among groups. Compared with the other FR groups, the acetate percentage was lower in the FR + MTS group, whereas the propionate percentage was lower in the FR + PMS group. The butyrate percentage was lower in the FR group than in the C group, whereas the butyrate percentage was higher in the FR + MTS and FR + PMS groups than in the FR group. The SCFA pool calculated as the total SCFA concentration per total digesta mass was higher in the FR + MTS and FR + PMS groups than in the FR group.

### 2.4. Function of Internal Organs and Lipid Metabolism in Rats after 4 Weeks of Feeding

Markers of liver function, kidney function and lipid metabolism did not differ between the C group and the FR group, whereas some of these markers were significantly affected by the tested seeds (Table 4). Compared with the FR and FR + MTS groups, the liver mass was higher in the FR + PMS group, whereas the liver fat percentage and cholesterol content were lower in the FR + PMS group. The mass of the kidneys was also higher in the FR + PMS group than in the other FR groups. The malondialdehyde (MDA) content in the liver and kidney, as a marker of lipid peroxidation, did not differ among the individual FR groups. The aspartate transaminase (AST), alanine transaminase (ALT) and alkaline phosphatase (ALP) activities in the blood plasma were higher in the FR + PMS group than in the other FR groups. The plasma urea concentration was higher in the FR + PMS group than in the FR + MTS group, whereas both of these groups had urea concentrations comparable to that of the FR group. The plasma cholesterol concentration did not differ among the individual FR groups. The plasma triglyceride concentration was lower in the FR + MTS group than in the FR + PMS group, whereas both groups had comparable triglyceride concentrations with that of the FR group.

## 3. Discussion

The aim of the present study was to verify whether MTSs and PMSs provide essential dietary components, such as quality protein, fat and fibre and how their regular consumption affects the body. Typically, plants of the family *Asteraceae* are not used in the daily diet, but with some important exemptions, such as chicory, artichoke or wild sunflower [21]. Because both types of seeds are high in dietary fibre, which is an important factor in the prevention and management of obesity and obesity-related diseases [22], we used FR diets in this model study on rats. MTSs and PMSs contained 45% fibre 62% dietary fibre, respectively (see Materials and Methods, Table 5). Moreover, both MTSs and PMSs contained significant amounts of protein (14% and 12%, respectively). MTSs also contained a considerable amount of fat (25%), whereas PMSs had significantly less fat (10%). Additional details on the chemical composition of MTSs used in the present study have been previously reported [23].

According to a recent review by Moura et al. [19], one of the most interesting categories of alternative proteins includes those of plant origin, which can be used for nutrition and health. Currently, soybean is considered the most popular source of protein among plant foods, the value of which is high and even comparable to some animal proteins [24]. The seeds of milk thistle and pot marigold, representing herbs in the family *Asteraceae*, have not been widely considered a source of protein that can diversify and improve the daily diet. Interestingly, some authors reported MTSs as a coffee substitute [25], whereas others used them to formulate functional biscuits [6]. According to the results of the present study, both types of seed proteins were less digestible when consumed together with the FR diet than soybean protein, especially that of PMSs (Table 2). Nevertheless, the protein quality of the diet containing MTSs was closer to that of the diet containing soybean protein isolate (FR group), with particularly comparable nitrogen retention in the body, whereas the protein quality of the diet containing PMSs was much worse than that in the FR group. Because MTSs do not contain tryptophan [7,8], this result indicated a good mutual complementation of essential amino acids from MTSs and casein that accounted for approximately half of the protein in the individual diets. As a consequence, differences between the protein quality of the diet containing MTSs and the diet containing PMSs were to some extent reflected in the body weight gains of rats (Table 1). The body weight gains were comparable between rats fed dietary soybean protein isolate and dietary MTSs, whereas rats fed dietary PMSs had less body weight gain, including body lean mass and fat mass, which may in part be due to their slightly lower dietary intake throughout the experiment.

The present study confirmed a well-known inverse relationship between dietary fibre content and dietary protein utilisation by the body, as both protein digestibility and nitrogen retention were lower in the FR group than in the C group (Table 2). Knudsen et al. [26] identified the main cause of this phenomenon as a decrease in the energy value of the diet resulting from its higher fibre content and thus an increased use of absorbed proteins for caloric needs of the body. In the present study, this phenomenon was even more likely because both the control and FR diets were deliberately deficient in protein to maximise protein utilisation in rats. Importantly, the present study showed that the FR diet fed to the FR group limited putrefaction in the caecum, which is the main location of anaerobic bacteria in rats. Evidence of limited putrefaction was found in reduced concentrations of ammonia and some SCFAs (isobutyrate, isovalerate and valerate; Table 3), which are the major amino acid metabolites in the distal gut [27]. The limited putrefaction indirectly indicated that dietary protein was better absorbed in the FR group and did not reach the distal intestine in larger amounts as was the case in the C group fed a regular amount of dietary fibre (5% diet). Nevertheless, a reduction in protein utilisation in growing rats also depends on the type of dietary fibre, which can differentially increase the excretion of endogenous and faecal nitrogen [28].

The inclusion of both types of seeds into the FR diet significantly affected the caecal metabolism of rats due to their fibre fractions. Importantly, the difference in fibre content between MTSs and PMSs (45% vs. 62%, respectively; Table 5), in addition to the same level of tested proteins in individual diets, resulted in the lack of balance in terms of dietary fibre content between the FR + MTS and FR + PMS groups. In the FR + MTS group, the difference in fibre content had to be partially compensated with the addition of cellulose, as it was the source of dietary fibre in the C and FR groups (Table 5). Regardless, dietary fibre from both MTSs and PMSs was a good substrate for bacterial fermentation in the caecum, as indicated by higher levels of total SCFAs, most individual SCFA concentrations and the SCFA pool. SCFAs, mainly acetate, propionate and butyrate, are the major end products of dietary fibre fermentation in the distal intestine, and they are considered indirect nutrients for the body [29]. Interestingly, milk thistle fibre was well fermented to propionate at the expense of acetate, which confirmed previous findings of increased propionate production from milk thistle fibre in the distal gut of genetically obese rats [23]. Propionate fermentation is especially important, because after absorption into the bloodstream, this acid reaches the liver, thus decreasing lipogenesis [30]. The present study showed for the first time that the fibre of PMSs is prone to fermentation in the distal gut.

The FR diet fed to the FR group did not affect lipid metabolism or the function of internal organs, whereas the inclusion of PMSs in the diet significantly affected lipid metabolism and internal organ function (Table 4). The FR diet containing PMSs reduced hepatic cholesterol levels, hepatic triglyceride levels and body fat gain, which together may be considered beneficial changes in lipid metabolism, especially in the context of obesity and cardiovascular disease epidemics. The lowered dietary intake in rats fed PMSs (vs. FR group) may have played a role in these beneficial changes, as well as the fat fraction of PMSs (10%), which is rich in calendic acid [16]. Chardigny et al. [31] reported a reduction in body fat in mice fed a diet supplemented with calendic acid. In general, conjugated isomers of *α*-linolenic acid are known to stimulate the *β*-oxidation of fatty acids in the liver, thus improving lipid metabolism [18]. In contrast, dietary MTSs only lowered plasma triglyceride levels (vs. the FR + PMS group), which may have been partly due to their lipid fraction (25% seeds), including linoleic acid, and/or intensified propionate production in the distal intestine (Table 3). Silymarin found in MTSs is known as a cholesterol-lowering factor rather than a triglyceride-lowering factor [23,25]. In the present study, dietary PMSs unfavourably affected the function of internal organs, as indicated by enlarged liver and kidneys after 4 weeks of feeding, and these changes were accompanied by increased ALT, AST and ALP activities, as well as blood urea concentration (Table 4). As PMSs are rich in many bioactive compounds, it is difficult to speculate which compounds are responsible for these negative changes. However, calendic acid is rather excluded from the suspected compounds, because Pintea et al. [32] reported that pot marigold seed oil lowers transaminase activity and ALP in the blood of rats with liver damage. Thus, further studies are needed to determine the responsible compounds. Interestingly, another plant of the family *Asteraceae* whose extract improved lipid metabolism in a mice model of fatty live disease was varthemia; however, unlike PMSs, the liver disorders were also ameliorated in this case [33]. When compared with other seeds, chia seeds of the family *Lamiaceae*, which are rich in *α*-linolenic acid, also had beneficial effects on lipid metabolism and liver function in rats, as well as relatively good protein quality [34].

The results of the present study and other studies [23] suggest that MTSs, as an alternative source of plant protein, fermentable fibre and polyunsaturated fatty acids, could be considered in the development of new food products. Because MTSs are available on the market in native and defatted forms, they can be included in the daily diet not only as a source of silymarin, but also as a source of nutrients. However, their organoleptic properties may be a challenge and should be further investigated. In addition, PMSs could be considered a promising ingredient of dietary supplements supporting obesity reduction, but their negative impact on internal organs, including possible liver damage, should be thoroughly studied and eliminated, for example, by adjusting their daily intake. Because the dietary content of both seed types was high in the present study (up to 39% for PMSs), it is impossible to reproduce such conditions in humans. Therefore, future human studies should be focused on adjusting the contents of PMSs in the diet while maintaining their therapeutic effects and monitoring the side effects shown in this study. An important limitation of the present study is that dietary protein was half of the recommended amount for rats, partly due to the chemical composition of MTSs and PMSs. Therefore, the digestibility and utilisation of the seed proteins would not be as high if all dietary requirements were met. However, we introduced these restrictions because we wanted to maximise protein absorption and utilisation and thus better compare the biological quality of dietary seed proteins with dietary soybean protein, as a model plant source of this nutrient.

## 4. Materials and Methods

*Chemical composition of milk thistle seeds (MTSs) and pot marigold seeds (PMSs).* Whole milk thistle (*Silybum marianum* L.) seeds were purchased from Intenson Europe Ltd. (Karczew, Poland), and pot marigold (*Calendula officinalis* L.) seeds in the form of achenes were obtained from a local crop located in Chąśno Drugie (Łódź Province, Poland). The chemical composition of MTSs and PMSs was quantified in triplicate by an accredited testing laboratory (Nuscana, Mrowino, Poland) in accordance with Polish–European ISO standards, official procedures of AOAC or internal procedures, using commonly known methods. Briefly, the dry matter and ash content were determined using the gravimetric method after drying whole MTSs and PMSs at 105 °C and 525 °C (PN-EN 1135:1999), respectively. The total dietary fibre was determined using the enzymatic–gravimetric method (AOAC 991.43:1994). Crude protein was determined using the Kjeldahl method (PN-EN ISO 20483:2014-02), and crude fat was determined using the Soxhlet extraction method. The nitrogen-free extract was then calculated by subtracting water, ash, fibre, crude protein and crude fat from 100. The chemical composition of MTSs and PMSs, as well as the composition of the experimental diets, are shown in Table 5.

*Animals, diets and experimental design.* The feeding experiment was conducted on 28 5-week-old male Wistar rats allocated to 4 groups of 7 individuals each. The initial body weight of rats was comparable among all groups (Table 1). For 28 days, rats were fed a semipurified diet with a regular fibre proportion (5% cellulose, control group) or a modification of this diet that was rich in fibre (24% cellulose, FR group). Both control (C) and fibre-rich (FR, positive control) groups had the same dietary proportions of protein and fat (9% and 10%, respectively). In the other FR groups, the diet was modified by adding ground MTSs or PMSs (FR + MTS or FR + PMS group, respectively) at the expense of a plant protein source (soybean protein isolate) and parts of other ingredients. All diets fed to the FR groups had comparable percentages of protein, fat and fibre (9%, 10% and 24%, respectively), but they slightly differed in the percentages of carbohydrates due to differences in the content of corn starch and the chemical composition of the tested seeds. The total protein content in all groups was nearly half of the recommended content for growing rodents [35] to maximise the degree of its absorption and utilisation by rats and thus reliably assess its biological quality. The experimental design shown in Figure 1 allowed us to compare the nutritional value of milk thistle and pot marigold seed proteins and that of soybean protein, which is recognised as the most valuable plant source of this nutrient. The detailed composition of the diets, which were freely available to rats for the entire experimental period, is shown in Table 5. Both types of seeds were ground for 1 min at a temperature below 37 °C prior to their inclusion in the diet. After preparation, the diets were stored at 4 °C under limited access to oxygen. The rats were individually housed in plastic cages and a controlled environment (12 h light/dark cycle, temperature of 21 ± 1 °C, relative humidity of 55 ± 10% and 15 air changes per hour). The animal protocol for this experiment was in compliance with European guidelines for the care and use of laboratory animals and was approved by the Local Institutional Animal Care and Use Committee in Olsztyn, Poland (permission number: 37/2017).

**Table 5:** Composition of the diets.

Ingredient (g/100 g)	Group ^1^				
	C		FR	FR + MTS	FR + PMS
Casein ^2^	5.13		5.13	5.13	5.13
Rapeseed oil (canola type)	9.8		9.8	2.1	6.2
Soybean protein isolate ^3^	4.87		4.87	-	-
Milk thistle seeds (MTSs) ^4^	-		-	31.91	-
Pot marigold seeds (PMSs) ^5^	-		-	-	38.57
Corn starch	60.50		41.50	36.45	35.16
Sucrose	10		10	10	10
Cellulose	5		24	9.71	0.24
Mineral mix ^6^	3.5		3.5	3.5	3.5
Vitamin mix ^6^	1		1	1	1
Choline chloride	0.2		0.2	0.2	0.2

^1^ C, control group fed a diet containing soy protein isolate; FR, positive control group fed a fibre-rich diet containing soy protein isolate; FR + MTS, experimental group fed an FR diet containing milk thistle seeds; FR + PMS, experimental group fed an FR diet containing pot marigold seeds. ^2^ Composition (g/100 g): dietary fibre not detected; crude protein, 88.7; crude fat, 0.3; lactose, 0.1; ash, 2.0. ^3^ Composition (g/100 g): dietary fibre not detected; crude protein, 91.0; crude fat, 3.3, nitrogen-free extract, <1. ^4^ Composition (g/100 g): dietary fibre, 44.8; crude protein, 13.9; crude fat, 24.8; nitrogen-free extract, 5.2; ash, 4.2. ^5^ Composition (g/100 g): dietary fibre, 61.6; crude protein, 11.5; crude fat, 9.8; nitrogen-free extract, 6.8; ash, 4.6. ^6^ Recommended for the AIN-93G diet [35].

*Body composition analysis.* At the beginning and end of experimental feeding, the body lean, fat and free fluid masses of the rats were determined with time-domain NMR using a Minispec LF 90II analyser (Bruker, Karlsruhe, Germany). The body lean, fat and free fluid percentages and gains were then calculated based on the initial and final body weights of the animals. The method relies on transmitting various radio frequency pulses into soft tissues to reorient the nuclear magnetic spins of hydrogen and then to detect radio frequency signals generated by the hydrogen spins from these tissues. The contrast in relaxation times of the hydrogen spins found among adipose tissue, muscles and free water is then used to estimate their masses within the body.

*Nitrogen balance.* At the beginning of experimental feeding, nitrogen balance was determined in rats allocated into metabolic cages capable of separating urine and faeces. First, a 5-day preliminary period was introduced to adapt the gut microbiota to the diets. A 5-day balance period was then started, and all faeces and urine were collected and preserved once daily. At the end of the balance period, the nitrogen levels in pooled faeces and urine were determined for each rat according to the Kjeldahl method. Apparent protein digestibility and nitrogen retention were then calculated (Table 2) and used as criteria for the nutritional value of dietary protein.

*Biological specimens.* After 4 weeks of experimental feeding, the rats were anaesthetised with a mixture of xylazine and ketamine in physiological salt (10 mg and 100 mg/kg body weight, respectively). Each rat was then weighed, and the abdomen was cut open. Blood was collected from the vena cava into heparinised tubes. The blood was then centrifuged for 10 min at 380× *g* and 4 °C, and the obtained plasma was frozen until analysis. The caecum, liver and kidneys were then removed, weighed and frozen in liquid nitrogen or used for further procedures.

*Faecal biochemistry.* Samples of fresh caecal digesta were collected, and their pH values were measured using a microelectrode and a pH/ION metre (Model 301; Hanna Instruments, Italy). The ammonia concentration in the fresh caecal digesta was extracted, trapped in a solution of boric acid and then quantified using direct titration with sulfuric acid in Conway dishes according to the method described by Hofirek and Haas [36]. The short-chain fatty acid (SCFA) concentrations were determined in the caecal digesta after storage at −20 °C using a gas chromatograph (Shimadzu Co., Kyoto, Japan) and a capillary column (SGE BP21; 30 m × 0.53 mm; SGE Europe Ltd., Milton Keynes, UK) as previously described [37].

*Internal organ biochemistry.* Malondialdehyde (MDA) was determined in the liver and kidneys after storage at −20 °C using a procedure developed by Botsoglou et al. [38]. The MDA content was determined spectrophotometrically at 532 nm (Helios Omega UV-VIS; Thermo Scientific, Waltham, MA, USA) and was expressed as micrograms of malondialdehyde per gram of organ. Liver lipids were extracted according to the Folch method with previously described modifications [39]. Briefly, the liver slice was homogenised with a 2:1 mixture of chloroform–methanol using a homogeniser (IKA T25; IKA Co., Shanghai, China) followed by centrifugation at 15,000× *g* for 10 min. The supernatant was washed with 0.8 mL of distilled water, vortexed and centrifuged for 15 min (2500× *g*). After removing the upper phase, the lower phase containing lipids was evaporated under a nitrogen stream at 37 °C. The lipid fraction obtained in this way was then dissolved with chloroform, and cholesterol and triglyceride concentrations were determined spectrophotometrically in this solution using reagents from Alpha Diagnostics Ltd. (Warsaw, Poland).

*Blood plasma biochemistry.* The plasma concentrations of cholesterol, triglycerides, uric acid, creatinine and urea, as well as the plasma activities of aspartate transaminase (AST), alanine transaminase (ALT) and alkaline phosphatase (ALP), were determined using a biochemical analyser (Pentra C200; Horiba Ltd., Kyoto, Japan).

*Statistical analysis.* The results were expressed as the means ± standard errors of the mean (SEMs). For comparisons between the C group and the FR group, Student’s *t*-test was used, and *p* ≤ 0.05 was considered statistically significant. If the distribution was not normal, the Mann–Whitney *U* test was used (*p* ≤ 0.05). Moreover, one-factor analysis of variance (ANOVA) and Duncan’s *post hoc* test were used to determine significant differences among the individual FR groups at *p* ≤ 0.05. If the ANOVA was not homogenous, one-factor Kruskal–Wallis ANOVA by rank was used, followed by Dunn’s Bonferroni-corrected *post hoc* test (*p* ≤ 0.05). All calculations were performed using Statistica version 13.1 (StatSoft Corp., Kraków, Poland).

## 5. Conclusions

The results of the present study indicate that certain seeds of the family *Asteraceae* provide dietary components of varied nutritional quality to rats. MTSs and PMSs are a rich source of dietary fibre, and the fermentability of the fibre is high and comparable between the seeds. In conditions of protein deficiency, unlike PMSs, MTSs are a relatively good source of protein, the quality of which does not significantly differ from that of soybean protein, when both are consumed in combination with animal protein. Further, dietary PMSs reduce body weight and lipid accumulation in the body, but they negatively affect the function of internal organs in rats. The obtained results may support further research on the possibility of introducing MTSs and PMSs into the human diet, as a source of nutrients and fermentable fibre, or on PMSs as a therapeutic agent in the management of obesity. Research should include the verification of their organoleptic properties, the adjustment of their daily intake and the elimination of possible side effects.

## Figures and Tables

**Table 1:** Dietary intake, body weight and body composition of rats after 4 weeks of feeding with a fibre-rich (FR) diet containing milk thistle seeds (MTSs) or pot marigold seeds (PMSs) ^1^.

	Group ^2^				
	C		FR	FR + MTS	FR + PMS
Initial body weight (g)	142 ± 2.62		141 ± 4.01	141 ± 2.05	142 ± 3.71
Initial fat (%)	13.5 ± 0.578		12.2 ± 0.563	12.4 ± 0.586	13.5 ± 0.702
Initial lean (%)	69.6 ± 0.620		70.3 ± 0.495	70.0 ± 0.628	69.3 ± 0.663
Initial free fluids (%)	3.83 ± 0.108		4.20 ± 0.139	3.95 ± 0.125	3.92 ± 0.074
Dietary intake (g/day)	18.6 ± 0.362		18.9 ± 0.301 ^ab^	19.4 ± 0.115 ^a^	15.7 ± 0.901 ^b^
Final body weight (g)	281 ± 4.55 *		266 ± 3.09 ^a^	267 ± 1.52 ^a^	222 ± 10.8 ^b^
Final fat (%)	21.4 ± 1.02		19.1 ± 0.892	17.3 ± 1.02	19.1 ± 0.560
Final lean (%)	63.1 ± 0.805		64.4 ± 0.746	65.5 ± 0.869	64.4 ± 0.558
Final free fluids (%)	4.35 ± 0.037		4.32 ± 0.062	4.49 ± 0.104	4.19 ± 0.102
Body weight gain (g)	139 ± 5.99 *		125 ± 1.46 ^a^	126 ± 2.85 ^a^	79.3 ± 8.97 ^b^
Fat gain (g)	40.9 ± 3.47		33.5 ± 1.86 ^a^	28.8 ± 2.89 ^ab^	23.0 ± 2.34 ^b^
Lean gain (g)	78.9 ± 3.21		72.5 ± 2.06 ^a^	76.5 ± 1.89 ^a^	44.0 ± 5.65 ^b^
Free fluid gain (g)	6.83 ± 0.267		5.60 ± 0.152 ^a^	6.42 ± 0.441 ^a^	3.67 ± 0.416 ^b^

^1^ Values are means ± SEMs (*n* = 7). ^2^ C, control group fed a diet containing soy protein isolate; FR, positive control group fed a fibre-rich diet containing soy protein isolate; FR + MTS, experimental group fed an FR diet containing milk thistle seeds; FR + PMS, experimental group fed an FR diet containing pot marigold seeds. * *p* ≤ 0.05 vs. the FR group (Student’s *t*-test or Mann–Whitney *U* test). ^a,b^ Labelled means in a row without a common letter differed at *p* ≤ 0.05 (Duncan’s or Dunn’s *post hoc* test).

**Table 2:** Nitrogen balance, protein digestibility and nitrogen retention in rats fed a fibre-rich (FR) diet containing milk thistle seeds (MTSs) or pot marigold seeds (PMSs) ^1^.

	Group ^2^				
	C		FR	FR + MTS	FR + PMS
Nitrogen					
Intake (mg/5 days)	1361 ± 49.5		1376 ± 10.5	1510 ± 1.04	1373 ± 78.5
In faeces (mg/5 days)	121 ± 8.22 *		157 ± 2.27 ^b^	326 ± 4.62 ^a^	370 ± 18.5 ^a^
In faeces (%N intake)	8.93 ± 0.646 *		11.4 ± 0.204 ^c^	21.6 ± 0.307 ^b^	27.0 ± 0.633 ^a^
In urine (mg/5 days)	397 ± 9.81 *		494 ± 24.9	468 ± 30.0	473 ± 12.3
In urine (%N intake)	29.6 ± 1.70 *		35.9 ± 1.74	31.0 ± 1.98	35.1 ± 1.98
Total digested protein (g/5 days)	7.75 ± 0.311		7.62 ± 0.070 ^a^	7.40 ± 0.030 ^ab^	6.27 ± 0.386 ^b^
Apparent protein digestibility ^3^ (%)	91.1 ± 0.646 *		88.6 ± 0.204 ^a^	78.4 ± 0.307 ^b^	73.0 ± 0.633 ^c^
Total retained nitrogen (mg/5 days)	8463 ± 56.5		725 ± 23.7 ^a^	716 ± 31.4 ^a^	530 ± 60.1 ^b^
Apparent nitrogen retention ^4^ (%)	61.5 ± 2.04 *		52.7 ± 1.68 ^a^	47.4 ± 2.09 ^a^	37.9 ± 2.46 ^b^

^1^ Values are means ± SEMs (*n* = 7). ^2^ C, control group fed a diet containing soy protein isolate; FR, positive control group fed a fibre-rich diet containing soy protein isolate; FR + MTS, experimental group fed an FR diet containing milk thistle seeds; FR + PMS, experimental group fed an FR diet containing pot marigold seeds. ^3^ Calculation: (protein intake−faecal protein)/protein intake × 100. ^4^ Calculation: (nitrogen intake−faecal nitrogen−urinary nitrogen)/nitrogen intake × 100. * *p* ≤ 0.05 vs. the FR group (Student’s *t*-test or Mann–Whitney *U* test). ^a,b,c^ Labelled means in a row without a common letter differed at *p* ≤ 0.05 (Duncan’s or Dunn’s *post hoc* test).

**Table 3:** Caecal metabolism in rats after 4 weeks of feeding with a fibre-rich (FR) diet containing milk thistle seeds (MTSs) or pot marigold seeds (PMSs) ^1^.

	Group ^2^				
	C		FR	FR + MTS	FR + PMS
Mass of empty segment (g/100 bw)	0.165 ± 0.005 *		0.209 ± 0.020	0.222 ± 0.008	0.241 ± 0.011
Digesta mass (g/g tissue)	3.25 ± 0.119 *		4.08 ± 0.226 ^b^	4.24 ± 0.319 ^b^	6.08 ± 0.540 ^a^
pH of digesta	7.72 ± 0.057		7.80 ± 0.136	7.56 ± 0.041	7.61 ± 0.153
Ammonia (mg/g digesta)	0.283 ± 0.008 *		0.140 ± 0.018	0.170 ± 0.007	0.195 ± 0.029
SCFA concentration (µmol/g digesta)					
Acetate	51.9 ± 2.16 *		27.9 ± 3.68 ^b^	45.4 ± 2.02 ^a^	44.1 ± 3.17 ^a^
Propionate	10.8 ± 0.583 *		5.89 ± 0.242 ^c^	9.91 ± 0.428 ^a^	7.20 ± 0.237 ^b^
Isobutyrate	1.06 ± 0.050 *		0.605 ± 0.074	0.741 ± 0.043	0.622 ± 0.045
Butyrate	8.79 ± 1.24 *		2.62 ± 0.560 ^b^	8.14 ± 0.643 ^a^	7.10 ± 1.68 ^a^
Isovalerate	0.988 ± 0.061 *		0.511 ± 0.081	0.591 ± 0.038	0.581 ± 0.067
Valerate	1.06 ± 0.155 *		0.553 ± 0.046 ^b^	0.838 ± 0.038 ^a^	0.778 ± 0.074 ^a^
Total SCFA ^3^	74.6 ± 3.31 *		38.1 ± 4.48 ^b^	65.6 ± 2.66 ^a^	60.3 ± 5.12 ^a^
Total PSCFA ^4^	3.11 ± 0.188 *		1.67 ± 0.186	2.17 ± 0.085	1.98 ± 0.159
SCFA proportion (% total conc.)					
Acetate	69.7 ± 1.16		72.5 ± 1.19 ^a^	69.2 ± 0.881 ^b^	73.4 ± 0.891 ^a^
Propionate	14.5 ± 0.382		16.4 ± 1.35 ^a^	15.2 ± 0.648 ^a^	12.2 ± 0.638 ^b^
Butyrate	11.7 ± 1.32 *		6.55 ± 0.893 ^b^	12.4 ± 0.734 ^a^	11.0 ± 1.57 ^a^
SCFA pool (µmol/total digesta mass)	114 ± 10.9		84.1 ± 10.4 ^b^	164 ± 12.8 ^a^	198 ± 28.5 ^a^

^1^ Values are means ± SEMs (*n* = 7). ^2^ C, control group fed a diet containing soy protein isolate; FR, positive control group fed a fibre-rich diet containing soy protein isolate; FR + MTS, experimental group fed an FR diet containing milk thistle seeds; FR + PMS, experimental group fed an FR diet containing pot marigold seeds. ^3^ Short-chain fatty acids. ^4^ SCFAs of putrefactive origin as the sum of isobutyrate, isovalerate and valerate. * *p* ≤ 0.05 vs. the FR group (Student’s *t*-test or Mann–Whitney *U* test). ^a,b,c^ Labelled means in a row without a common letter differed at *p* ≤ 0.05 (Duncan’s or Dunn’s *post hoc* test).

**Table 4:** Internal organ functions and lipid metabolism in rats after 4 weeks of feeding with a fibre-rich (FR) diet containing milk thistle seeds (MTSs) or pot marigold seeds (PMSs) ^1^.

	Group ^2^				
	C		FR	FR + MTS	FR + PMS
Liver					
Mass (g/100 bw)	3.29 ± 0.104		3.24 ± 0.081 ^b^	2.94 ± 0.091 ^b^	3.87 ± 0.202 ^a^
Fat (% liver)	10.4 ± 0.486		9.66 ± 0.330 ^a^	10.0 ± 0.573 ^a^	8.20 ± 0.255 ^b^
Triglycerides (mg/g liver)	5.37 ± 0.588		5.12 ± 0.277	5.57 ± 0.382	4.78 ± 0.438
Cholesterol (mg/g liver)	1.34 ± 0.091		1.39 ± 0.118 ^a^	1.65 ± 0.094 ^a^	1.11 ± 0.049 ^b^
MDA (µg/g liver) ^3^	0.600 ± 0.048		0.561 ± 0.016	0.570 ± 0.035	0.644 ± 0.052
Kidneys					
Mass (g/100 bw)	0.647 ± 0.018		0.626 ± 0.011 ^b^	0.649 ± 0.021 ^b^	0.734 ± 0.018 ^a^
MDA (µg/g kidney) ^3^	3.39 ± 0.656		1.95 ± 0.662	2.42 ± 0.265	3.28 ± 0.185
Plasma					
AST (U/L) ^4^	50.5 ± 2.81		46.7 ± 3.60 ^b^	57.4 ± 4.51 ^b^	82.6 ± 10.1 ^a^
ALT (U/L) ^4^	26.9 ± 1.53		23.8 ± 2.13 ^b^	25.3 ± 1.24 ^b^	82.7 ± 13.3 ^a^
ALP (U/L) ^4^	255 ± 15.6		228 ± 23.9 ^b^	190 ± 11.4 ^b^	346 ± 39.0 ^a^
Uric acid (µmol/L)	25.4 ± 1.09		25.4 ± 2.02	28.0 ± 3.32	49.4 ± 19.2
Creatinine (µmol/L)	18.7 ± 2.91		12.4 ± 1.85	11.3 ± 3.59	18.4 ± 2.08
Urea (mmol/L)	1.89 ± 0.245		1.85 ± 0.071 ^ab^	1.58 ± 0.153 ^b^	2.56 ± 0.322 ^a^
Cholesterol (mmol/L)	1.78 ± 0.065		2.02 ± 0.132	1.79 ± 0.069	1.98 ± 0.057
Triglycerides (mmol/L)	2.02 ± 0.254		2.47 ± 0.286 ^ab^	1.47 ± 0.263 ^b^	3.13 ± 0.534 ^a^

^1^ Values are means ± SEMs (*n* = 7). ^2^ C, control group fed a diet containing soy protein isolate; FR, positive control group fed a fibre-rich diet containing soy protein isolate; FR + MTS, experimental group fed an FR diet containing milk thistle seeds; FR + PMS, experimental group fed an FR diet containing pot marigold seeds. ^3^ Malondialdehyde. ^4^ AST, aspartate transaminase; ALT, alanine transaminase; ALP, alkaline phosphatase. ^a,b^ Labelled means in a row without a common letter differed at *p* ≤ 0.05 (Duncan’s or Dunn’s *post hoc* test).

## Data Availability

Not applicable.
